# Utilisation of services provided at an outreach healthcare facility and its associated factors among residents in a coastal taluk of southern Karnataka: A cross-sectional study

**DOI:** 10.51866/oa.513

**Published:** 2024-04-27

**Authors:** Deepak Sudhakaran, Sneha Deepak Mallya, Akhilesh Kumar Pandey, Ranjitha S Shetty

**Affiliations:** 1 MD in Community Medicine, Department of Community Medicine, Kasturba Medical College, Manipal, Manipal Academy of Higher Education, Manipal, Karnataka, India. Email: ranjitha.shetty@manipal.edu; 2 Social Works and Community Engagement, Pallium India, Thiruvananthapuram, Kerala, India.; 3 MD in Community Medicine, Department of Community Medicine, Kasturba Medical College, Manipal, Manipal Academy of Higher Education, Manipal, Karnataka, India.; 4 PhD, Department of Community Medicine, Kasturba Medical College, Manipal, Manipal Academy of Higher Education, Manipal, Karnataka, India.

**Keywords:** Health service, Facilities and services utilization, Primary healthcare

## Abstract

**Introduction:**

Understanding the patterns of utilisation of primary health services can help to improve service delivery and utilisation, thereby reducing common morbidities in the community. The study aimed to assess the patterns of utilisation of services provided at an outreach healthcare centre.

**Methods:**

A community-based survey was conducted among families residing in the field practice area of an outreach centre for more than a year. Data were collected using a questionnaire administered to adults aged >18 years. Collected data were entered into and analysed using the Statistical Package for the Social Sciences version 16.0.

**Results:**

Approximately 65.1% of the respondents were aged 31–59 years, and 67.4% were women. Among 126 surveyed households, 50.7% had utilised services from the outreach centre. The facilitators of utilisation among 64 households included proximity to their area of residence (90.6%) and availability of good-quality services (40.6%). The most common barriers included a lack of awareness (30.9%) and inconvenient timings (18.2%) of the healthcare centre. The respondents aged <18 years (odds ratio [OR]=7.64; 95% confidence interval [CI]=4.37–13.37) and >45 years (OR=2.65; 95% CI=1.57–4.47) had higher odds of utilising services than those aged 18–45 years. The female respondents (OR=2.89; 95% CI=1.86-4.51) were more likely to utilise services than the male respondents.

**Conclusion:**

Creating awareness regarding the outreach healthcare centre and designing services based on the observed needs of the community can further improve the utilisation of services provided at the healthcare centre.

## Introduction

India has a population of 1.38 billion people and has one of the fastest-developing economies worldwide.^1^ However, having a healthy population is a pre-requisite to ensuring that the country can continue the same trajectory of growth and development, with studies suggesting that economy and health are bidirectionally related.^2,3^ With a rapid epidemiological and demographic transition, India is likely to witness a large increase in non-communicable diseases (NCDs). To cope with this burden, the healthcare system needs to adopt and provide a range of services encompassing preventive, promotive, curative and palliative care. India has a robust healthcare system with three tiers of healthcare infrastructure catering to the needs of people from all strata of society. These tiers include subcentres (SCs), primary healthcare centres (PHCs), community healthcare centres, subdistrict hospitals, district hospitals, medical colleges and centres of excellence providing various kinds of services at each level.^4^ However, the National Sample Survey in 2014 revealed that only 11.5% and 3.9% of the rural and urban populations in the country had accessed the large network of primary healthcare facilities (HCFs), respectively.^5^

Udupi, being one of the forward districts of Karnataka, a southern state of India, in terms of literacy and socioeconomic status (SES), has a well-developed healthcare system. Its healthcare system provides healthcare through a large network of public and private sector hospitals. Prior studies have shown that the morbidity trends due to NCDs in this region are substantially comparable to the national trends.

A private medical college situated in the district has been providing health services to the population of the district as well as neighbouring districts and states. The rural and semi-urban populations residing in the region receive primary health services through its five peripheral healthcare centres affiliated with the medical college. Despite being private HCFs, these centres provide either free or highly subsidised services. Providing all services that a government-run HCF offers may not be feasible in this setting. Although many services have been proposed to be implemented by upgraded primary HCFs, a healthcare centre will be successful only if the services being offered to a community are based on needs. Hence, this study aimed to assess the current utilisation patterns of services provided at one of these centres located in a semi-urban area, understand the needs of the community and determine the facilitators and barriers to availing such services.

## Methods

A community-based survey was conducted in the urban field practice area of the department of Community Medicine of a private medical college in southern Karnataka, India, from 2018 to 2020. The field practice area covers a population of about 10,463, which spreads across 2260 households. The study area is equipped with a good network of HCFs, with services that are easily accessible and affordable being provided through a PHC from the public sector and an outreach centre affiliated with the department of Community Medicine. Additionally, there are various private clinics and nursing homes including two Ayurvedic facilities. Within a 10-km radius, there are also four tertiary HCFs that provide health services to people in this region. The outreach centre provides a range of health services, such as maternal and child care, screening and management of NCDs including common cancers and geriatric care. The centre facilitates the delivery of basic health services to the community through regular clinics. Consultations, basic essential drugs and immunisations are provided with follow-up visits at no cost, and laboratory investigations are offered at highly subsidised charges. Maternal and child health (MCH) services include antenatal, intranatal and postnatal services; immunisation; management of childhood illness; and health education services. A health insurance scheme is provided to residents in the field practice area of the department of Community Medicine for health services at subsidised rates across secondary and tertiary care hospitals affiliated with the medical college. The centre also offers physiotherapy, occupational therapy and optometry services as allied health components.

The study proposal was approved by the Institutional Ethics Committee (IEC 574/2018). The study included both men and women aged ≥18 years from families residing in the field practice area of the outreach centre for more than 1 year. Based on an estimated 50% of the population having utilised at least one or more health services from the outreach healthcare centre, a precision of 10% and a non-response rate of 25%, the estimated sample size was 125 households.

There are 18 localities in the field practice area of the HCF. The number of households in each locality was obtained from the data maintained at the HCF obtained through regular field activities. From each locality, the number of houses to be included was estimated using the probability proportional to size method to ensure the representativeness of the study area. Thereafter, households were selected using simple random sampling. One eligible respondent from each family was recruited after obtaining written informed consent. A pre-designed semi-structured questionnaire was administered by the interviewer to collect sociodemographic data as well as other information required to assess the patterns of utilisation of health services from the outreach centre. Various facilitators and barriers to availing such health services were ascertained. The SES of the surveyed families was assessed using the Modified BG Prasad Scale.^6^

Herein, an acute illness was defined as an illness that is self-limiting and lasts for a short duration. Minor acute illnesses included some of the commonest problems noted in general practice, such as upper respiratory tract infections or skin rashes.^7^ An acute major illness was defined as an acute illness that is self-limiting or requires treatment and may present as an acute exacerbation of an underlying chronic illness, such as myocardial infarction or diabetic coma, or a sudden onset of a previously undiagnosed condition, such as epilepsy or stroke, or an acute emotional or psychological problem.^7^ A chronic illness was defined as an illness persisting for more than 3 months.^8^

Quantitative data were analysed as frequencies and proportions using the Statistical Package for the Social Sciences for Windows, Version 16.0. SPSS Inc. Released 2007, Chicago, IL, USA). The chi-square test was used to establish associations between the sociodemographic and other factors affecting the utilisation of health services. Univariable logistic regression was applied to identify the factors affecting the utilisation rate, expressed as odds ratios (ORs) with 95% confidence intervals (CIs). All variables that were included in the univariable analysis were included in multivariable logistic regression to determine the factors independently associated with the utilisation of health services provided by the outreach centre. A P-value of <0.05 was considered statistically significant.

## Results

A total of 126 households were surveyed, and the utilisation pattern of health services among 578 individuals was assessed. Approximately two-thirds of the respondents (65.1%) were aged 31–59 years, and the majority (67.4%) were women. Nearly three-fourths of the respondents (73.8%) were married. Further, 65.8% had 5–12 years of schooling, while only 3.9% were illiterate. Almost half of the respondents (46.8%) were homemakers, whereas 31.7% had unskilled/semiskilled jobs. The majority of the respondents (75.4%) were Hindus, and over one-third belonged to nuclear families. Approximately 86.5% of the surveyed households belonged to the middle socioeconomic class based on their Modified BG Prasad Scale scores. Of the 578 respondents, nearly a quarter (22.4%) had one or more chronic illnesses, with hypertension (14.7%) and diabetes (6.7%) being the most common types. Approximately 23.5% of the respondents had at least one acute illness within the past 5 years.

Among the 126 surveyed households, 64 (50.7%) had utilised services from the outreach centre. There were a total of 578 members currently living in the 126 households, among whom 23.01% (n=133) had utilised one or more services provided at the outreach centre. Of the 178 individuals who reported a history of chronic illness, only 7.8% had utilised services from the outreach centre, and 8.9% were exclusively availing services from a government HCF. The majority of the chronic illnesses were treated at a private HCF, with other private hospitals being utilised the most (33.1%), followed by private clinics (25.2%), private medical college hospitals (17.4%), nursing homes (7.3%), government PHCs (6.7%) and government hospitals (2.2%). Similarly, most acute minor and major illnesses were treated at private HCFs (85.4% and 94.7%, respectively), and less than 10% of the population had availed services from government sources. However, as a facility that provides primary care services, the outreach centre was utilised for acute minor illnesses only (5.6%).

The majority of the respondents were aware of the health insurance scheme provided by the outreach centre (82.5%). The respondents were also aware of screening and management of NCDs (65.8%), child health services (61.9%), maternal health services (45.2%), laboratory services (38.1%), cancer screening and referral services (34.1%), basic ophthalmological services (18.2%), health education sessions (18.2%), physiotherapy and occupational therapy services (9.5%) and family planning services (1.5%). The source of information regarding services provided at the outreach centre was auxiliary nurse midwives during their household visits (90.6%), family members (9.4%) and friends (6.2%).

The most commonly availed services from the outreach centre were child health services (39.8%), followed by screening and management of NCDs (38.3%), health education sessions (20.3%), laboratory services (14.2%), cancer screening and referral services (6.7%), maternal health services (6.02%), physiotherapy and occupational therapy services (3.7%), basic ophthalmological services (3.7%) and dental services (0.7%).

The facilitators to service utilisation were assessed among 64 families who had utilised any services in the last 5 years. The majority quoted the centre being in proximity to their area of residence (90.6%) as the reason for utilisation, followed by availability of good-quality services (40.6%). Conversely, the barriers to utilising services from the outreach centre were assessed among all households since every household had some or other barriers to mention when they were asked about the non-utilisation of specific services. The most common barriers identified were a lack of awareness about the services provided at the centre (30.9%) and inconvenient timings (18.2%) of the centre (Table 1).

**Table 1:** Facilitators and barriers to availing services from the outreach healthcare centre among the surveyed families.

Facilitators (n=64)	Frequency (%)
Proximity to home	58 (90.6)
Good-quality service	26 (40.6)
Consultations and medications at no cost	6 (9.4)
Reputation of healthcare providers	4 (6.2)
Other reasons^*^	11 (17.0)
**Barriers (n=126)**	
Lack of awareness	39 (30.9)
Inconvenient timing	23 (18.2)
Doctor-related reasons (e.g. unavailability of doctors round the clock or different doctors on each consultation)	9 (7.1)
Non-availability of desired services at the facility	8 (6.3)
Other reasons^#^	11 (8.7)

* Availability of good laboratory services at subsidised rates, availment of insurance scheme, short waiting period and good staff

# Availability of selected free drugs, far location, unawareness of the centre, poor experience at the centre, poor trust in doctors from a private setup and fear of knowing the diagnosis

**Table 2** shows that in the univariable analysis, age, sex and type of family were significantly associated with the utilisation of services from the outreach centre. All variables were included in the multivariable analysis, which showed that only age and sex were significantly associated with the availment of services from the outreach centre. The respondents aged <18 years (OR=7.64; 95% CI=4.37–13.37) and >45 years (OR=2.65; 95% CI= 1.57–4.47) had higher odds of utilising services from the outreach healthcare centre than those aged 18–45 years. The female respondents (OR=2.89; 95% CI=1.86-4.51) were more likely to utilise such services than the male respondents.

**Table 2:** Association between the sociodemographic characteristics and utilisation of services from the outreach healthcare centre among the respondents (N=578).

Characteristics	Utilisers (n=133) Frequency (%)	Nonutilisers (n=445) Frequency (%)	Univariable analysis		Multivariable analysis	
			Crude OR (95% CI)	P-value	Adjusted OR (95% CI)	P-value
	<18 years	55 (41.35)	62 (13.94)	**7.26 (4.25-12.38)**	**<0.01**	**7.64 (4.37-13.37)**	**<0.01**
Age^*^	18–45 years	28 (21.05)	229 (51.46)	1		1	
	>45 years	50 (37.59)	154 (34.60)	**2.66 (1.60-4.40)**	**<0.01**	**2.65 (1.57-4.47)**	**<0.01**
Sex^#^	Male	42 (31.58)	241 (54.16)	1		1	
	Female	91 (68.42)	204 (48.84)	**2.56 (1.70-3.86)**	**<0.01**	**2.90 (1.86-4.51)**	**<0.01**
	Hindu	90 (67.67)	340 (76.40)	0.82 (0.38-1.81)	0.63	0.96 (0.41-2.24)	0.92
Religion	Muslim	34 (25.56)	77 (17.30)	1.37 (0.59-3.22)	0.47	1.66 (0.65-4.26)	0.29
	Christian	9 (6.77)	28 (6.30)	1		1	
	Upper class	8 (6.02)	34 (7.64)	1		1	
SES	Middle class	121 (90.98)	389 (87.42)	1.32 (0.60-2.93)	0.49	1.26 (0.52-3.01)	0.61
	Lower class	4 (3.01)	22 (4.94)	0.77 (0.21-2.88)	0.70	1.09 (0.26-4.56)	0.91
	Nuclear	33 (24.81)	135 (30.34)	1		1	
Type of family	Three-generation	46 (34.59)	110 (24.72)	**1.71 (1.02-2.86)**	<0.05	1.46 (0.83-2.59)	0.19
	Joint	54 (40.60)	200 (44.94)	1.11 (0.68-1.79)	0.69	0.93 (0.54-1.59)	0.79

* Age adjusted for the remaining four variables: sex, religion, SES and type of family

# Sex adjusted for the remaining four variables: age, religion, SES and type of family

CI, confidence interval; OR, odds ratio; SES, socioeconomic status

Among the 126 surveyed households, 86 respondents (68.2%) desired to avail additional services at the outreach centre (Table 3). With respect to healthcare provider-related suggestions, over half of the respondents wanted a full-time doctor at the centre (52.3%). With respect to the infrastructure of the centre, 8.1% suggested that ambulance services should be available. The health services desired by the respondents included creating awareness about the service availability (34.8%) and providing delivery services at the centre (23.3%).

**Table 3:** Additional health services at the outreach healthcare centre desired by the respondents (n=86).

Services		Frequency (%)
**Healthcare provider-related services**	Availability of full-time doctors	45 (52.3)
	Availability of specialists	9 (1G.5)
	Availability of more experienced doctors	8 (9.3)
	Availability of the same doctor on every visit	6 (7.G)
**Infrastructure-related services**	Need for ambulance services	7 (8.1)
	Need for a spacious facility	2 (2.3)
	Need for more physiotherapy equipment	2 (2.3)
	Creation of awareness in the community about the centre	3G (34.9)
	Provision of delivery services	2G (23.3)
	Provision of radiological services	13 (15.1)
**Other related health services**	Provision of emergency services	13 (15.1)
	Need for more drugs	7 (8.1)
	Need for in-patient services	4 (4.6)
	Provision of home care services	4 (4.6)
	Immediate availability of laboratory reports at the centre and organisation of more health camps	3 (3.5)

## Discussion

In this study, 64 of the 126 surveyed households (50.7%) and 133 of the 578 individual members (23.01%) had utilised services from the outreach centre. A large proportion of the utilisers (41.3%) were aged <18 years, which could be due to the availability of free immunisation and growth monitoring services. Further, the geriatric age group (20.3%) utilised health services more often mainly due to the availability of regular NCD screenings, with subsidised examinations and essential drugs being provided at no cost. This finding may also be due to the proximity of the centre to their homes and inability to travel because of frailty and chronic conditions. In contrast, over half of the non-utilisers (51.5%) were aged 18–45 years. Conversely, in a study conducted in Uttar Pradesh, a northern state of India, 45.3% of non-utilisers of services provided at a PHC were aged above 50 years.^8^ In the present study, the respondents aged <18 years (OR=7.64; 95% CI=4.37–13.37) and >45 years (OR=2.65; 95% CI=1.57-4.47) were more likely to utilise health services. A study performed in southern Nigeria also found that with increasing age, respondents were more likely to have positive health-seeking behaviours.^9^

Women constituted 68.4% of the utilisers in the present study. This proportion is higher than that in a study conducted in Uttar Pradesh (53.5%).^10^ The present study found that the female respondents (OR=2.90; 95% CI= 1.86–4.51) had higher odds of utilising health services from the centre. Similarly, a study performed in South Africa showed that women had a 2.18-fold higher odds of utilising health services.^11^

In another study conducted in Nigeria, it was found that with advancing age, women were more likely to use family planning services. Further, the utilisation of child health services increased with income but decreased with age. The availment of delivery services from institutions improved with better education, occupation and income.^12^ A secondary analysis of National Family and Health Survey-3 data from India revealed that the standard of living was significantly associated with the utilisation of health services, with people having a low standard of living having a 1.53-fold higher odds of utilising services from a HCF.^13^

In the present study, 42.1% of the utilisers belonged to the middle socioeconomic class. In a study conducted in a north Indian state, a larger proportion of utilisers (71%) was from the middle class.^8^ Although the present study showed an association between the type of family and utilisation of health services, it was not statistically significant (OR=1.46; 95% CI=0.83–2.59). Hindus constituted the majority of the utilisers (67.6%), followed by Muslims (25.5%) and Christians (6.7%), representing the general distribution of religions in the region. Among the utilisers, 40.6% were from joint families, while 34.6% were from three-generation families; these proportions are smaller than that reported in a study conducted in Delhi, wherein 61.9% of utilisers belonged to nuclear families.^10^ Further, a study performed in southern Nigeria showed that individuals who received up to secondary education were 0.46 times less likely to have positive health-seeking behaviours.^9^

The majority of the respondents in the present study utilised private HCFs for acute and chronic illnesses and MCH services. Conversely, a study conducted in rural areas of Punjab found that a little over 50% of 660 participants had made use of a public HCF that provided outpatient and in-patient care services. In addition, private facilities were frequented rather than their public counterparts for treating NCDs.^14^ However, a study performed in southern India showed that more than half of individuals (56.2%) received screening services, and 40.7% received treatment for NCDs from public HCFs.^15^ In the present study, the proximity to home was quoted as the most common reason (90.6%) for availing services from the centre, followed by good-quality service (40.6%) and free treatment (9.3%). In contrast, a study conducted in Uttar Pradesh demonstrated that 20% of utilisers had mentioned free treatment as a reason for availing services from a local PHC.^8^ Similarly, a study conducted in Kerala reported that good behaviour of healthcare workers, improved infrastructure and clean premises and subsidised laboratory services were quoted as facilitators to using services provided at a family healthcare centre.^16^ In another study, conducted in Mumbai, trust over providers (31.5%), proximity to home (20%), staff availability (12.7%), good-quality treatment (10.2%) and time-efficient services (8%) were cited as reasons for the utilisation of services from an urban healthcare centre.^17^

Herein, the most common barriers to the utilisation of services from the centre were a lack of awareness (30.9%) and inconvenient timing (18.2%). Likewise, a study conducted in Uttar Pradesh reported that 29.8% of non-utilisers cited unawareness of services being provided by a local PHC.^8^ Another study from the same region showed that long distance to the HCF (63.5%), non-availability of medicines (20.5%), inconvenient timing of the HCF (16.5%) and unavailability of a full-time doctor (16%) were barriers to the utilisation of services offered by PHCs and SCs.^18^ The same study also showed long waiting hours at the HCF (56.4%) and long distance from residence to the HCF (27.2%) to be the most common barriers among urban residents.^18^ A study from West Bengal reported free medicine supply (29.6%), good treatment (27%), proximity (18.4%), 24/7 availability of doctors (14.5%) and low cost of services (10.5%) as reasons for availing health services from government facilities and good treatment as the main reason for availing services from private facilities.^19^

### Limitations

The utilisation rates of health services from the outreach centre might have been over- or underestimated, as only self-reported information from the respondents from each family was collected.

## Conclusion

Age and sex are significantly associated with the utilisation of services provided at the outreach centre. Proximity to home and availability of good-quality services are the commonly cited facilitators to the utilisation of services from the healthcare centre. Poor awareness about available health services and inconvenient clinic timings resulted in underutilisation of services provided at the centre.

## Recommendations

Creating awareness regarding the outreach healthcare centre and its services and having periodic feedback from the community can improve the utilisation of health services provided at the centre. The utilisation of health services can further improve if such services are designed based on the felt needs of the community. Further research involving focus group discussions or in-depth interviews should be conducted to generate qualitative data that may be essential for understanding the perceptions of the community with respect to healthcare utilisation in the area.

## Data sharing statement

The study data are available from the corresponding author and can be provided on request.

**How does this paper make a difference in general practice?**

- Understanding the utilisation patterns of health services provided at primary healthcare facilities can help to improve the quality of service delivery as well as suggest measures to overcome barriers to seeking care.
- This information can also aid in providing services based on the felt needs of the community and thereby enhancing service utility among the community.
- Improved utilisation of health services can indirectly help to reduce the burden of diseases in the community.
- The findings of the study are useful for primary care physicians in developing strategies and improving the quality of services.

## References

[1] Department of Economic and Social Affairs PDU (2020). World Population Prospects 2019..

[2] Verma CS, Usmani G (2019). Relationship between health and economic growth in India.. Indian J Hum Dev..

[3] Amiri A, Gerdtham U-G (2013). Impact of maternal and child health on economic growth: new evidence based Granger causality and DEA analysis.. http://devdata.worldbank.org/wdi2011.htm.

[4] Chokshi M, Patil B, Khanna R (2016). Health systems in India.. J Perinatol..

[5] Ved RR, Gupta G, Singh S (2019). India's health and wellness centres: realizing universal health coverage through comprehensive primary health care.. WHO South East Asia J Public Heath..

[6] Mathiyalagen P, Davis P, Sarasveni M (2021). Updated BG Prasad Socio-Economic Classification: the 2020 update.. Indian J Pediatr..

[7] Jones R, White P, Armstrong D (2010). Managing acute illness.. An inquiry into the quality of general practice in England..

[8] Rajpurohit AC, Srivastava AK, Srivastava VK (2013). Utilization of primary health centre services amongst rural population of northern India - some socio-demographic correlates.. Indian J Community Health..

[9] Adam V, Aigbokhaode A (2018). Sociodemographic factors associated with the healthcare-seeking behavior of heads of households in a rural community in southern Nigeria.. Sahel Med J..

[10] Arora M, Koshy G, Gangadharan V (2019). Determinants of utilization of health services.. Int J Community Med Public Health..

[11] Abaerei AA, Ncayiyana J, Levin J (2017). Health-care utilization and associated factors in Gauteng province, South Africa.. Glob Health Action..

[12] Agunwa CC, Obi IE, Ndu AC (2017). Determinants of patterns of maternal and child health service utilization in a rural community in south eastern Nigeria.. BMC Health Serv Res..

[13] Dey DK, Mishra V (2014). Determinants of choice of healthcare services utilization: empirical evidence from India.. Indian J Community Health..

[14] Singh T, Bhatnagar N, Singh G (2018). Healthcare utilization and expenditure patterns in the rural areas of Punjab, India.. JFamily Med Prim Care..

[15] Bhagyalakshmi CK, Kodali PB (2019). Utilization of noncommunicable disease services provided by public health facilities in Kasaragod, Kerala.. Arch Med Health Sci..

[16] Vijayan SM, Puliyakkadi S, Chalil S (2021). Facilitators and barriers of service utilization: perspectives of stakeholders in a family health center of central Kerala - a qualitative study.. Indian J Public Health..

[17] Patil S, Parbhankar S, Bansode-Gokhe S (2016). Study of health seeking behavior and its determinants among attendees of urban health center, Dharavi, Mumbai, India.. Int J Community Med Public Health..

[18] Shukla V, Agarwal M, Idris MZ, Ahmed N, Gupta P (2018). Utilization of public health care facilities in Lucknow district.. Int J Community Med Public Health..

[19] Ray SK, Basu SS, Basu AK (2011). An assessment of rural health care delivery system in some areas of West Bengal-an overview.. Indian J Public Health..

